# ’A contradiction between our state and the tobacco company’: conflicts of interest and institutional constraints as barriers to implementing Article 5.3 in Bangladesh

**DOI:** 10.1136/tobaccocontrol-2021-057142

**Published:** 2022-01-25

**Authors:** S M Abdullah, Tracey Wagner-Rizvi, Rumana Huque, Sushama Kanan, Samina Huque, Rob Ralston, Jeff Collin

**Affiliations:** 1 Department of Economics, University of Dhaka, Dhaka, Bangladesh; 2 ARK Foundation, Dhaka, Bangladesh; 3 Global Health Policy Unit, The University of Edinburgh School of Social and Political Science, Edinburgh, UK

**Keywords:** public policy, tobacco industry, low/middle income country

## Abstract

**Introduction:**

Bangladesh has not yet adopted measures to implement Article 5.3 of the WHO Framework Convention on Tobacco Control. The National Tobacco Control Cell (NTCC) has drafted a guideline for implementation, but progress has stalled amid high levels of tobacco industry interference in public policy. This paper examines the barriers to minimising industry interference in a context of close relationships between government officials and tobacco companies.

**Methods:**

In-depth interviews were conducted with government officials, representatives from civil society, think tank and media organisations, and academic researchers. The data were analysed using a ‘3 Is’ framework developed within the political sciences, emphasising the interactive role of *ideas, interests* and *institutions* in policy change.

**Results:**

The findings indicate that policy *ideas* about protecting public health policy making from tobacco industry interests are largely restricted to the Ministry of Health and Family Welfare, and the NTCC specifically. Both individual and institutional conflicts of *interest* emerge as key barriers to progress to minimising industry interference and for tobacco control governance more broadly. The data also suggest that development of an Article 5.3 guideline has been shaped by the perceived interests of political actors and institutions, and the *institutional* position of the NTCC, constrained by limits on its resources, authority and isolation from other ministries.

**Conclusion:**

NTCC’s initiatives towards implementing Article 5.3 constitute an important opportunity to address conflicts of interest that restrict tobacco control in Bangladesh. Progress in minimising industry interference is essential to realising the commitment to being smoke free by 2040.

## Introduction

In April 2020, during a COVID-19 lockdown affecting industries across Bangladesh, the then Secretary at the Ministry of Industries instructed officials to ensure the ongoing production and distribution of tobacco products.^1–3^ This instruction was given shortly after receiving a letter from the managing director of British American Tobacco Bangladesh (BATB) emphasising the status of cigarettes as an ‘essential commodity’ under legislation that predates Bangladesh independence, the Control of Essential Commodities Act 1956 (East Pakistan Act). This letter requested that the Secretary issue such an order to ‘facilitate our effort to ensure an **uninterrupted flow of revenue in the government exchequer**’^4^ (*emphasis in original*), and a similar letter was received from the Japan Tobacco International-owned United Dhaka Tobacco. The BATB letter in effect constituted an exchange between tobacco company colleagues, with the then Secretary having been appointed to the BATB board as a non-executive director in 2018, one month after his appointment to the Ministry position.^5 6^ When a new Secretary was appointed to the Ministry in May 2020, the new occupant assumed a position on the BATB board the following month (on the same day that his predecessor stood down).^7 8^

This episode illustrates the extent to which the tobacco industry, and BATB in particular, remains closely entwined with government in Bangladesh. In June 2020, BATB’s listing of its six non-executive or independent directors comprised senior officials from the Ministry of Agriculture, Ministry of Industries, Ministry of Finance, the state-owned central bank (Bangladesh Bank) and investment bank (Investment Corporation of Bangladesh, ICB), as well as a government-appointed representative reflecting its shareholding in the company (reported at 0.64% plus 5.75% via ICB).^9 10^ Having ratified the WHO Framework Convention on Tobacco Control (FCTC) in 2004, Bangladesh has an obligation to implement Article 5.3 and its measures to protect public health policies relating to tobacco control ‘from commercial and other vested interests of the tobacco industry in accordance with national law’.^11^ Civil society monitoring reports have indicated comparatively high levels of tobacco industry interference in public policy,^12 13^ and the country’s 2020 FCTC implementation report stated that no measures had been adopted to protect against industry interference in policy nor recent progress made in implementing Article 5.3.^14^

Bangladesh’s previous submission in 2018, by contrast, had reported that national guidelines on implementing Article 5.3 would ‘be finalized very soon and … disseminated to all concerned’.^15^ The task of developing such guidance falls to the National Tobacco Control Cell (NTCC). Established in 2007 as the national coordinating mechanism mandated by the Tobacco Products (Control) Act 2005, the NTCC sits within the Ministry of Health and Family Welfare (MoHFW) and is headed by the Additional Secretary (Public Health and World Health) of the Health Services Division, with day-to-day coordination by a Joint Secretary. Established with technical support from WHO and The Union (formerly the International Union Against Tuberculosis and Lung Disease), with financial backing from the Bloomberg Initiative to Reduce Tobacco Use, this small unit contains three programme officers, one account and logistics assistant and an office assistant.^16 17^ The NTCC’s work on managing industry interference has included drafting a code of conduct for its officials and a national guideline for Article 5.3 implementation, but progress towards publication and endorsement of these documents has stalled.^14^

In this paper, we examine the barriers to advancing efforts to implement Article 5.3 and its guideline recommendations,^18^ in a context of close interlocking relationships^19 20^ between the Bangladeshi government and tobacco industry. It draws on the ‘3 Is’ framework developed within the political sciences, which theorise the interactive role of *ideas, interests* and *institutions* in policy change.^21–27^

Drawing on in-depth, semistructured interviews, we first explore the extent of support for Article 5.3 and its idea of protecting public health policies from industry interference through minimising engagement with the tobacco industry.^23 28^ In analysing interests, we focus specifically on the significance of conflicts of interest, exploring how policy contexts are shaped by their operation both at individual level (eg, where primary obligations as a civil servant may conflict with secondary objectives as a tobacco company director)^29^ and at institutional level, characterised by tensions between governmental commitments to health goals and close relationships with, and investments in, the tobacco industry.^30 31^ In this context, we then assess the institutional position of the NTCC, characterised by limited resources, capacity and authority, and consider how such constraints interact with conflict of interest to impede progress on Article 5.3.

## Methods

This paper draws on 15 in-depth, semistructured interviews with government officials from the MoHFW (n=1), Ministry of Finance (n=2) and Ministry of Commerce (n=1), in addition to representatives from civil society (n=3), think tanks (n=4) and media organisations (n=1), and academic researchers with experience of tobacco control debates (n=3). SMA and RH developed an initial list of interviewees based on publicly available information, grounded in familiarity with tobacco control policy developments in Bangladesh. Interviewee selection was also guided by ‘snowball’ sampling^32^ using networks and suggestions made by other interviewees. While this appears a relatively small sample, it represents mid-ranking and senior government officials with experience of government–industry engagement, but also reflects the limited number of individuals working on tobacco control policy within the NTCC and MoHFW.

Interviews were conducted by SMA, SK and SH between February–July 2020 and April–May 2021. This extended period of fieldwork reflects the impact of the COVID-19 pandemic and mitigation measures, including lockdown and travel restrictions. The pandemic similarly impacted on the availability of interviewees (particularly of government officials) amid increased work pressures and changing roles. Interviews varied between 40 and 66 min (averaging 55 min) with almost all conducted using teleconferencing software due to COVID-19 restrictions. Interviewees were asked to review a consent form that allowed interviews to be recorded and for the data to be used in research outputs, with interviewees providing verbal consent at the start of teleconference calls.

Interviews were semistructured and followed a topic guide addressing four thematic areas: FCTC Article 5.3 and the development of policies for its implementation; interaction between government and the tobacco industry; coordination on tobacco control between different ministries; and tobacco industry activities during COVID-19. All interviews were conducted in Bengali, transcribed and anonymised and then translated to English. Interview transcripts were analysed in NVivo V.12 using a thematic coding framework that was developed iteratively through descriptive and then conceptual coding. Interview transcripts were dual coded by researchers at ARK Foundation (SK, SMA, SH) and Edinburgh (TW-R, RR), with input from RH and JC.

## Results

### Article 5.3 and its ideas: limited awareness of protecting against industry interference

The data suggest that familiarity with Article 5.3 and its constitutive idea of protecting public health policy making from tobacco industry interests was limited to the Ministry of Health, and that detailed knowledge was concentrated in the NTCC. Officials in other ministries were seen as either being completely unaware of Article 5.3 or else having very limited knowledge.

Within the MoHFW, engagement with the FCTC, familiarisation with Article 5.3’s ideas and NTCC’s work in developing guidance were seen as reflected in changes in perspective and practices. Recalling the process of developing the 2005 Act, one health advocate described how the MoHFW had still operated under the assumption that the tobacco industry should be engaged in the policy process:

At that time no one was so vocal about Article 5.3… [We] saw that when the Ministry of Health did the committee, it invited the tobacco company to the committee. They wanted the British American Tobacco Company to cooperate with the government in enacting the law, and in a meeting, we saw that they were present.

By contrast, in the context of more recent efforts to advance tobacco control, interview data indicate that Article 5.3 norms and practices have been broadly adopted among health officials. This has been led by the work of the NTCC but seems to extend across the MoHFW, presented by one government official as being in marked contrast to the continuing ability of the tobacco industry to access other ministries:

The tobacco company has no direct relationship, connection or interaction with the NTCC or with the Department of Health Services or with the [MoHFW]. But the tobacco company has direct connections with various ministries of the government or the institutions under the ministry.

Interviewees were agreed that knowledge and understanding of Article 5.3 was very low in other ministries. This was depicted as being a function of tobacco control itself not being seen as relevant beyond the MoHFW, with one government official describing colleagues as having ‘no need to know’ about practices that are not perceived as being ‘within a domain you’re personally interested in or within the domain of your work responsibility’. A civil society representative attributed low awareness in other ministries to seeing tobacco control responsibilities as confined to health and failure to recognise that Article 5.3 implied commitment across government departments:

[It] is a matter of the whole state and it needs a comprehensive effort to control it, they don’t understand this thing. They know [little] about Article 5 and they think it seems to apply to the Ministry of Health, not to them. […] There is a lack of knowledge and they don’t know clearly about the obligations they have.

Given this lack of familiarity and patterns of close relationships with the tobacco industry, raising the idea of restricting industry interactions appears on occasion to have represented a culture shock. An interviewee from a think tank described the incredulous response of Agriculture officials to a letter from a Non-government organization expressing concern about transparency:

They said to us that—‘An organization has given us a letter, see how funny it is! They are saying we should not have a relationship with the tobacco industry, is it possible? Suppose my brother works in the tobacco industry and it’s his daughter’s marriage. Will I not go to his daughter’s wedding?’

### Conflicts of interest: individual and institutional

The data highlighted broad concerns about the tensions arising from government officials simultaneously holding positions in BATB, highlighting conflicts between duties to the public and to shareholders and between national and personal interests. One interviewee from a think tank noted that ‘when I am the … director of a company then the responsibility of developing that company falls on us’, and other interviewees including a media representative and a government official also highlighted the opportunities for policy influence arising from such senior appointments:

How can government secretaries perform their duties day after day on the board of a private organization? They are [the] Secretary of Industries, Secretary of Agriculture, one is from the Ministry of Finance, ICB. That is, those who will make an impact in policy making.

[W]hat happens here if you sit on the board, you get a good amount of remuneration, a director’s fee etc, [which] means it can sometimes affect policy.

Interviewees cited the favourable taxation structure enjoyed by tobacco companies^33^ as indicative of the policy significance of such ties, including via the perceived influence of the outgoing chair of the National Revenue Board who had been an independent director of BATB prior to his appointment.^34–37^

Beyond the direct role conflicts of senior government officials on the BAT board, interview data drew attention to the tobacco industry’s range of pervasive links across the policy elite in Bangladesh. One researcher described financial incentives for diverse actors (‘Member of Parliament, high rank government officers, … and so many other people’), while conflicts could also arise via links with family members. One government official described having seen the son of a prominent politician emerging from a car with a BAT logo ahead of a meeting:

Now the son of the [leading politician] has come in the car of BAT and there we will ask the [same politician] about the harmful aspects of tobacco cultivation. What will he understand, what will he decide? His son is coming out from the car of BAT, in the car of a tobacco company. This is the kind of influence. Influence is not only through government officials, but also through political leaders or their family members.

The embedded and extensive nature of such links is also viewed as deterring colleagues who are aware of Article 5.3 commitments from raising issues surrounding industry interference. A civil society interviewee noted a tendency to overlook issues that could cause tensions with peers or senior colleagues: ‘They think about their personal career because they think it’s what they need. They think it’s going to get him in trouble, he will get pressure from other ministries.’

The speed with which the Ministry of Industries issued its letters of support for protecting tobacco as an essential good during the COVID-19 crisis was presented as indicative of the reach and significance of industry influence in government. One government official noted that the letter was issued at a time when all government offices beyond MoHFW were closed during this lockdown, yet ‘the Ministry of Industries received a letter from BAT on Thursday. In the wake of that letter, the letter [of instructions] was issued on Friday’. The official marvelled that the letter, which could not be issued without the Secretary’s approval, could be written and approved so quickly and issued on the weekly holiday: ‘It’s a miracle event to me. I have been in a government institution for three years, [and] there is no way to issue such a letter. This means that the influence of the tobacco company is strong.’

These individual conflicts arise from close institutional relationships that reflect both the extent of BATB’s strategic links with key ministries and the extent of the government’s stake in BATB as both a shareholder and perceived source of revenue. Leading government officials are seen as attractive as board members precisely because of these institutional roles; one think tank interviewee noted of the board of directors, ‘the finance secretary, he is there because of his post. The agriculture secretary … is there for his post.’

One health advocate described the government’s shareholding and the positions on the BATB board of senior government officials as meaning that ‘[w]e have a contradiction between our state and the tobacco company’. Such tensions were articulated with reference to incoherence across the government’s health and economic goals. In 2016, the prime minister announced a vision of Bangladesh as being tobacco free by 2040,^38 39^ while the government has a long-term strategic economic development plan of becoming a high-income country by 2041^40^ that places a strong emphasis on increasing foreign direct investment.^41^ The stated commitment to a tobacco-free future has not yet been accompanied by a clear tobacco control strategy for achieving it, and interviewees saw this as being ‘subordinate to economic considerations’. One interviewee from the media noted that ‘[e]ven after such a big announcement, they are not as concerned about it as [they are] concerned about tax or policy making. Especially in the case of tax’.

A civil society representative highlighted tensions between the government’s stake in BATB and its obligation under Article 18 of the Bangladesh constitution to improving nutrition and public health as the primary duties of the state (and specifically to ‘adopt effective measures to prevent the consumption … [of] drugs which are injurious to health’).^42^

[You] have to prohibit harmful things. […] As tobacco is harmful so according to constitutional obligation the government will regulate that. So, when the government is part of that, it doesn't matter whether it’s 10 percent or 1 percent. […] That is conflicted with the constitution.

The call to jettison the state’s stake in BATB to tackle such conflicts was echoed by several interviewees, including a media representative who stated that industry interference ‘can only be stopped when the government withdraws the company’s shares’ and a think tank representative who cited government shares among the ‘many big barriers here, [and] these have to be overcome’.

### Institutional constraints and the NTCC

In this complex context of conflicting interests, interview data suggest that the ability of the NTCC to address issues of tobacco industry interference is heavily circumscribed by inter alia limits in its resources and authority, and its isolation from other ministries in contrast with the seniority and reach of key BATB-linked government officials. The pressures within which the NTCC operates can be illustrated by the reaction to its efforts to intervene in the essential goods decision. The NTCC reportedly wrote to the Ministry of Industries to request the suspension of tobacco production and marketing for the duration of lockdown. According to one government official interviewed, the NTCC ‘wrote the letter after 5 pm, got the reaction within an hour, negative reaction. And the next day [they found] out that it [was] not received by the Ministry of Industry’. Pressure was described as having been applied from multiple departments and ministries, including from the highest levels. Leading authorities were described as noting both that ‘the economy is already under pressure’ and that ‘the tobacco company pays a lot of tax’, and as having questioned why the MoHFW had requested the suspension of tobacco production.

This episode seems consistent with broader opposition by ministries and government agencies to the NTCC’s efforts to accelerate tobacco control. One government official described, ‘when it comes to tobacco control policy, we see that the National Board of Revenue, the Ministry of Agriculture, the Ministry of Industries are some of the ministries that have obstructed [progress]. Absolutely discouraging it.’ Such dynamics help to understand the stalling of efforts to approve a draft guideline on restricting industry interference, with this official describing the process as stuck at a point whereby government adoption of the 5.3 guidelines as a legal framework ‘now requires an inter-ministerial meeting’. An interviewee from a think tank also highlighted the links between BATB and key ministries in explaining the impasse on the NTCC’s draft guideline, given the need to secure consensus across ministries:

If you want to do … the guideline, you have to take all the ministries. Because the Ministry of Industry is involved there, the Ministry of Agriculture is involved there. Those who are already on that board of directors have to do it, with everyone’s consent.

Although mandated by the Tobacco Control Law, headed by an Additional Secretary and knowledgeable about tobacco control, interview data consistently indicate that the NTCC does not have the authority or influence necessary to effect change. The NTCC is a small office and, according to one civil society interviewee, ‘[t]hey do not have the capacity to work at the policy-making stage. Therefore, they do not have the capacity to implement what they know.’ While the NTCC has received funding via Bloomberg, one civil society interviewee highlights the restricted status of team members this supports; ‘they are not employees of the government. They have no voice since they are not government officials. They have knowledge but they have no chance to practice it.’

The NTCC is viewed as being comparatively isolated within the MoHFW, and therefore restricted in its capacity to drive broader engagement with Article 5.3 ideas across the government, as might be expected of an institution mandated to serve as the national coordinating mechanism in accordance with the FCTC. One government official described the NTCC as operating ‘under the Department of Public Health … [with] a direct and indirect connection to the Department of Healthcare’, but as having ‘no direct relationship with the other departments like health education and family welfare. Absolutely no communication’.

The NTCC is depicted as being largely powerless to prevent or intervene in interactions between the tobacco industry and other ministries or in international fora. One government official described how on many occasions ‘representatives of tobacco companies go to many ministries; they can access very easily’ with the NTCC typically being unable to act, often only learning about such engagements after the event. The NTCC is also described as being unable to stop interactions that it is aware of, particularly where ‘there is an involvement with any influential officer or any senior top-level official’.

The very seniority of the government officials recruited to the BATB board itself serves to inhibit more junior officials from advancing the NTCC’s agenda. The same official described the significance of BATB appointing the Secretary of a key government department as a *de facto* veto:

The secretary of the ministry is the administrative head of all. […] So, there is no chance for an additional secretary, a joint secretary or a deputy secretary to go against the secretary. So, if the Secretary is on the Board of BAT, […] there is no opportunity to do anything.

Interview data suggest that the inability to make progress towards approving the draft code of conduct and Article 5.3 implementation guideline is entangled with broader difficulties in advancing a national tobacco control strategy. One government official described how the NTCC had hoped to secure cross-ministerial approval for a national strategy and ‘then proceed with the 5.3 guidelines of the FCTC article. But because our policy is stuck, it is also delayed’.

## Discussion

The policy-making process and outcome in the specific context of a COVID-19 lockdown highlights the broader challenges of seeking to make progress in managing tobacco industry interference in Bangladesh. This account of barriers to securing approval for the NTCC’s draft code of conduct and a guideline for Article 5.3 implementation demonstrates how limited progress in establishing norms and practices can be explained via extensive conflicts of interest and the institutional constraints within which the NTCC operates. In terms of the ‘3 Is’ framework, the inability to effectively establish the *idea* of protecting public health policy making from tobacco industry interests reflects the interplay of powerful *interests*, operating via key individuals and across key ministries and agencies, and imbalances of power, authority and resources across key *institutions*.^21–27^ These interest-based and institutional factors have constrained the impact of ideas on tobacco control policy change in Bangladesh.

The degree of interpenetration between BATB and senior government officials, epitomised by the composition of the company’s board, is striking in international comparative terms and raises profound questions about the governance of conflicts of interest that extend beyond tobacco control. Such relationships are analysed in the management and business study literatures via the concept of *interlocking directorates*,^19 20^ referring to the links established ‘when a person affiliated with one organization’ (in this case a ministry in the Bangladesh government) ‘sits on the board of directors of another organization’^20^ (BATB). This literature draws attention to the significance of the roles that strategically selected independent or non-executive directors can perform, including providing access to high-level expertise and advice, enhancing communications with stakeholders and external organisations, informing strategic development, enhancing legitimacy with key audiences and securing access to (and facilitating the support of) policy makers.^19 19 43 44^ The account presented above is consistent with the positions of key government officials on the BATB board of Bangladesh being significant barriers to advancing tobacco control in general and Article 5.3 implementation in particular.

The literature on the benefits to firms of having the expertise of politicians and policy makers in the boardroom centres on studies of those who have previously occupied such roles.^43 44^ In the context of a pressing challenge such as the COVID-19 lockdown, it seems reasonable to hypothesise that being able to appoint current occupants of key roles would be even more valuable. The rapidity with which the Ministry of Industries responded to BATB concerns, considered remarkable for any circumstances let alone amid lockdown, seems consistent with it. These role conflicts for senior officials point to the inadequacy of existing rules of conduct for government servants in Bangladesh. Issued in 1979, these do not address conflicts of interest and so cannot guard effectively against government officials making inappropriate use of their expertise and access to advance private or corporate interests that may conflict with national goals.^45^ This highlights the extent to which Article 5.3 guidelines engage with much wider issues of governance around transparency, accountability and conflict of interest^18^; their implementation may be more effectively advanced in synergy with the establishment of broader norms and practices consistent with tobacco-specific concerns.^46^

The interview data suggest that individual conflicts of interest arising from senior officials in key ministries simultaneously serving as BATB directors exacerbate problems in addressing institutional tensions between government mandate and company interests. Decision-making in the context of COVID-19 is striking for the extent to which industry claims of significance and value to the Bangladesh economy appear to have been endorsed at the highest levels of government. The case for tobacco control as enhancing the country’s economic and sustainable development objectives is well established,^33 47 48^ yet industry-affiliated individuals and ministries continue to present company interests as aligned with national objectives.

These conflicts are clearly important in understanding the institutional constraints to which the NTCC is subject. But the interview data also highlight the significance of its comparative marginalisation within the government. The NTCC was mandated to act as the national coordinating mechanism for tobacco control in accordance with the FCTC, but its limited engagement with other ministries and even with other parts of the MoHFW exacerbates challenges of developing effective coordination that are familiar in other contexts.^49–51^ This highlights the importance of strengthening the NTCC to advance Article 5.3 implementation and strengthen tobacco control in Bangladesh.

At an international level, improved implementation of Article 5.3 has been identified as the single highest priority for advancing tobacco control.^52^ The severity of the levels of industry interference outlined above, consistent with civil society monitoring and prior studies,^12 13 39 53^ strongly suggests that this priority is particularly acute in the case of Bangladesh. Similarly, difficulties in promoting multisectoral coordination across government departments and agencies are central to the challenge of advancing implementation of Article 5.3, both in Bangladesh and internationally.^54^ While the barriers outlined here are significant, advancing the work of the NTCC in developing a code of conduct and guideline for implementation also constitutes an important opportunity to drive forward the tobacco control agenda in Bangladesh. Taking advantage of this opportunity appears as a prerequisite for significant progress towards the government’s stated ambition of becoming tobacco free over the next two decades, and as coherent with the vision of sustainable development towards becoming a high-income country.

**What this paper adds**

- High levels of tobacco industry interference in policy in Bangladesh have been reported, but efforts to advance Article 5.3 implementation have not previously been analysed.
- Close relationships between the government and the tobacco industry are epitomised by the presence of senior government officials on the board of British American Tobacco Bangladesh and by the state holding shares in the company. Associated conflicts of interest present significant barriers to advancing tobacco control, particularly with regard to Article 5.3.
- The National Tobacco Control Cell (NTCC) is mandated to act as the national coordinating mechanism for tobacco control in accordance with the Framework Convention on Tobacco Control. Its capacity to fulfil this role is restricted by limits in its resources, capacity, authority and engagement with other ministries.
- Advancing the work of the NTCC towards implementing Article 5.3 in Bangladesh is essential to advancing both tobacco control and sustainable development.

## Data Availability

No data are available. Not applicable.
